# Caracterización fenotípica de la retinitis pigmentaria asociada a sordera

**DOI:** 10.7705/biomedica.6129

**Published:** 2022-05-01

**Authors:** Ángela Camila Paredes, Greizy López, Nancy Gelvez, Marta Lucía Tamayo

**Affiliations:** 1 Instituto de Genética Humana, Pontificia Universidad Javeriana, Bogotá, D.C., Colombia Pontificia Universidad Javeriana Pontificia Universidad Javeriana Bogotá, D.C. Colombia

**Keywords:** retinitis pigmentaria, fenotipo, pérdida auditiva, trastornos sordoceguera, síndromes de Usher, diagnóstico clínico., Retinitis pigmentosa, phenotype, hearing loss, deaf-blind disorders, Usher syndromes, clinical diagnosis

## Abstract

**Introducción.:**

El síndrome de Usher es una alteración genética caracterizada por la asociación de retinitis pigmentaria y sordera. Sin embargo, hay casos con familias en las cuales, a pesar de presentarse dicha asociación, no se puede diagnosticar un síndrome de Usher ni ninguno otro.

**Objetivo.:**

Reevaluar fenotípicamente a 103 familias con diagnóstico previo de posible síndrome de Usher o retinitis pigmentaria asociada con sordera.

**Materiales y métodos.:**

Se revisaron las historias clínicas de 103 familias con un posible diagnóstico clínico de síndrome de Usher o retinitis pigmentaria asociada con sordera. Se seleccionaron las familias cuyo diagnóstico clínico no correspondía a un síndrome de Usher típico. Los afectados fueron valorados oftalmológica y audiológicamente. Se analizaron variables demográficas y clínicas.

**Resultados.:**

Se reevaluaron 14 familias cuyo diagnóstico clínico no correspondía al de síndrome de Usher. De las familias con diagnóstico inicial de síndrome de Usher típico, el 13,6 % recibieron uno posterior de “retinitis pigmentaria asociada con sordera” de “otro síntoma ocular asociado con hipoacusia’,’ o en forma aislada en una misma familia, de “retinitis pigmentaria” o “hipoacusia’.’

**Conclusiones.:**

Es fundamental el estudio familiar en los casos en que la clínica no concuerda con el diagnóstico de síndrome de Usher típico. En los pacientes con retinitis pigmentaria asociada con sordera, el diagnóstico clínico acertado permite enfocar los análisis moleculares y, así, establecer un diagnóstico diferencial. Es necesario elaborar guías de nomenclatura en los casos con estos hallazgos atípicos para orientar a médicos e investigadores en cuanto a su correcto manejo.

## Abstract (en)

**Introduction:**

There are several syndromes that associate retinitis pigmentosa with deafness or hearing loss. The most frequent is Usher syndrome, a genetic disorder of autosomal recessive inheritance, which, in some cases, is accompanied by vestibular dysfunction. However, there are cases of families that despite having retinitis pigmentosa associated with deafness, cannot be classified as Usher or other syndromes due to additional findings.

**Objective:**

To reassess the phenotypes of 103 families previously diagnosed as possible Usher syndrome and/or retinitis pigmentosa associated with deafness.

**Materials and methods:**

We conducted a descriptive and retrospective study by reviewing the medical records of 103 families with a probable clinical diagnosis of Usher syndrome and/or retinitis pigmentosa associated with deafness. Families whose clinical diagnosis did not correspond to the typical Usher syndrome were selected and evaluated ophthalmologically and audiologically. Demographic and clinical variables were analyzed.

**Results:**

We selected and then reevaluated 14 families and 55 individuals as they did not correspond to a clinical diagnosis of Usher syndrome; 13.6% of the families initially considered to have typical Usher syndrome were later diagnosed with retinitis pigmentosa associated with deafness, another ocular symptom associated with hearing loss, retinitis pigmentosa, or isolated hearing loss in the same family.

**Conclusions:**

Family studies are essential in cases where the symptoms do not match the typical Usher’ syndrome. In the cases of retinitis pigmentosa associated with deafness, a correct clinical diagnosis allows for focusing on the molecular analyses to establish a differential diagnosis. The need for nomenclature guidelines on these atypical findings is relevant to aid physicians and researchers in the best approach to these cases.

La retinitis pigmentaria es una enfermedad genética que se caracteriza por pérdida de agudeza visual, ceguera nocturna y reducción del campo visual. El examen de fondo de ojo revela acumulación de pigmento con un aspecto de espículas de hueso ^1^^,^^2^. Su diagnóstico se basa en la documentación de la pérdida progresiva de la función de los fotorreceptores, lo que se evidencia con un electrorretinograma, pruebas de campo visual y fotos de fondo de ojo mediante angiografía con fluoresceína ^3^^,^^4^. Actualmente, se conocen más de 80 genes asociados con esta enfermedad, los cuales se clasifican según el patrón de herencia ^5^^,^^6^.

La hipoacusia neurosensorial, por su parte, puede ser causada por un síndrome, una infección, un trauma o una malformación que altere el oído interno o la vía auditiva, o su funcionamiento, comprometiendo las células ciliadas cocleares y, consecuentemente, las neuronas ganglionares espirales ^7^. Aproximadamente, el 60 % de las hipoacusias son de origen genético y se han asociado con más de 50 genes ^8^.

La forma de asociación más común de retinitis pigmentaria e hipoacusia neurosensorial congénita es el síndrome de Usher, que es la causa más frecuente de sordoceguera en humanos ^9^^,^^10^, y que, según el tipo clínico, se asocia también con alteración de la función vestibular ^11^.

Es una condición de origen genético con un patrón de herencia autosómico recesivo, cuya prevalencia se calcula entre 3,5 y 6,2 casos por cada 100.000 habitantes ^11^. En Colombia, la frecuencia es de 3,2 por cada 100.000 habitantes: el 9,6 % con hipoacusia y el 10 % con ceguera ^12^^-^^14^.

Se han descrito tres tipos según los genes comprometidos y las manifestaciones clínicas, pero existen formas de la enfermedad que, debido al mecanismo de herencia, la historia natural de la enfermedad o algunas manifestaciones atípicas, no pueden catalogarse como síndrome de Usher ^10^^,^^15^. Usualmente, estos casos se clasifican como síndrome de Usher atípico y su confirmación requiere el estudio molecular, aunque en algunos se hallan signos que descartan la sospecha clínica ^16^.

El presente estudio descriptivo se hizo entre 2016 y 2017 en Bogotá, con el objetivo de revalorar fenotípicamente a 103 familias con un diagnóstico inicial de síndrome de Usher. En dicha revaloración, se encontraron 14 familias cuyo diagnóstico clínico no correspondía a un típico síndrome de Usher.

Se sabe que el diagnóstico clínico de las enfermedades genéticas es definitivo para su manejo y que una apropiada sospecha clínica permite solicitar oportunamente los estudios genéticos que permitan confirmar el diagnóstico. Sin claridad sobre el fenotipo, se dificulta la confirmación de la enfermedad y el inicio del tratamiento apropiado ^17^^,^^18^, lo que puede implicar una mayor morbilidad en algunos casos.

En este sentido, se concluyó que los médicos tratantes deben sospechar la presencia de dos condiciones genéticas independientes en un mismo individuo, las cuales simularían un síndrome específico, lo cual tiene un impacto en el enfoque diagnóstico que se adopte.

## Materiales y métodos

### Población de estudio

En un estudio descriptivo y retrospectivo, se revisaron las historias clínicas de 103 familias sin relación entre ellas y con un posible diagnóstico clínico de síndrome de Usher. Estas familias habían sido incluidas y valoradas en el marco del “Programa de atención integral a familias con enfermedades huérfanas con componente visual y auditivo” (AIVA) del Instituto de Genética Humana de la Pontificia Universidad Javeriana de Bogotá.

Cabe señalar que previamente se habían hecho los estudios moleculares de estas familias, los cuales incluyeron el análisis de ligamiento de los genes asociados con el síndrome de Usher y la secuenciación de Sanger de las mutaciones más frecuentemente relacionadas con este. En dichos estudios, no se identificaron mutaciones ni hubo evidencia de ligamiento con alguno de los genes estudiados.

De las 103 familias, 14 no presentaban el fenotipo del síndrome de Usher clásico. Se analizaron 55 individuos con retinitis pigmentaria asociada con sordera o aislada, o con hipoacusia no sindrómica. Los individuos afectados fueron valorados por un oftalmólogo especialista en retina. Se hizo el examen de fondo de ojo y se complementó con exámenes paraclínicos como electrorretinograma, campimetría y angiografía en los casos en que fue posible. Para valorar la pérdida auditiva, se practicó audiometría tonal y, en algunos casos, logoaudiometría y timpanograma.

### Recolección y análisis de datos

Se revisaron las historias clínicas de los 55 individuos pertenecientes a las 14 familias reclasificadas con el diagnóstico de retinitis pigmentaria asociada a sordera. Las variables analizadas fueron: edad al ingreso en el programa AIVA, lugar de nacimiento, sexo, edad de inicio del primer síntoma, edad de inicio de la hipoacusia, gravedad de la enfermedad y compromiso vestibular. La hipoacusia se clasificó según la edad de inicio en: congénita, desde el nacimiento; en lactantes, desde el mes de vida hasta los dos años; en preescolares, de 2 a 5 años de edad; en escolares, entre los 5 y los 11 años; en adolescentes, a partir de los 12 años; en adultos jóvenes, desde los 18 hasta los 29 años, y tardía, desde los 30 años.

Se diseñó una base de datos en Excel en la que se ingresaron variables como la edad de inicio de la retinitis pigmentaria y de la pérdida auditiva, los hallazgos oftalmológicos y el compromiso auditivo. Se aplicaron medidas de tendencia central y se calcularon las frecuencias relativas para cada una de las variables.

### Consideraciones éticas

El estudio fue aprobado por el Comité de Investigación y Ética de la Facultad de Medicina de la Pontificia Universidad Javeriana. Todos los individuos incluidos firmaron el consentimiento informado, aceptando la recolección de datos de la historia clínica y su publicación.

## Resultados

Se seleccionaron y reevaluaron 14 familias de las 103 analizadas, a las cuales no les correspondía un diagnóstico clínico de síndrome de Usher. Es decir, de las familias con diagnóstico inicial de síndrome de Usher típico, el 13,6 % recibieron uno posterior de “retinitis pigmentaria asociada con sordera” de “otro síntoma ocular asociado con hipoacusia’,’ o en forma aislada en una misma familia, de “retinitis pigmentaria” o “hipoacusia’.’ Se analizaron en total 55 individuos pertenecientes a las 14 familias.

El 43,6 % de los individuos estaba en un rango de edad entre los 40 y los 49 años en el momento de inicio del estudio, con un promedio de 50 años y una moda de 35, 46 y 47 años. De los 55 estudiados, 23 (42 %) eran de sexo femenino y 32 (58 %) de sexo masculino, con una relación hombre-mujer de 1,4:1,0 y sin mayor prevalencia según el sexo.

En cuanto a la distribución geográfica, las familias provenían de diferentes regiones del país, pero principalmente de Bogotá y de zonas aledañas como Fusagasugá. Sin embargo, también se encontraron familias provenientes de poblaciones lejanas no relacionadas, de los departamentos de Meta, Santander, Antioquía y Boyacá. El 29 % correspondía a familias consanguíneas.

Los 55 individuos valorados se dividieron según sus evaluaciones clínicas y oftalmológicas en tres grupos: el primero incluyó los casos de retinitis pigmentaria (ya sea aislada, asociada a sordera o asociada a otro síntoma); el segundo, a aquellos individuos con otro síntoma ocular aislado o asociado a sordera, y el tercero, a aquellos con hipoacusia. En el cuadro 1 se describen las frecuencias en cada caso.

**Cuadro 1:** Descripción global del compromiso visual y auditivo de los individuos afectados

Compromiso	n	%
Individuos con retinitis pigmentaria	33	60
Retinitis pigmentaria aislada	13	39,4
Retinitis pigmentaria más sordera *	14	42,4
Retinitis pigmentaria más otro síntoma	6	18,2
Individuos con otro síntoma ocular	9	16,4
Otro síntoma ocular aislado	3	33,3
Otro síntoma ocular más sordera *^^^	6	66,7
Individuos con hipoacusia	36	65,5
Aislada	13	36,1
Asociada a otra sintomatologia*	23	63,9

* Las personas que comparten la sintomatología visual y auditiva fueron contabilizadas en las categorías señaladas. ^ Se encontraron tres personas con retinitis pigmentaria y estrabismo asociados a sordera.

### Compromiso auditivo

En lo que respecta a la lateralidad de la hipoacusia, de un total de 36 individuos, el 8 % (3/36) tenía diagnóstico de hipoacusia unilateral; el 75 % (27/36) presentaba hipoacusia bilateral, y los datos del 17 % (6/36) de ellos no estaban disponibles.

**Figura 1:**
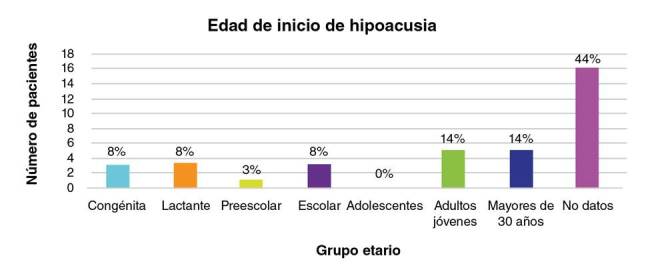
Distribución de la edad de inicio de la hipoacusia

En cuanto al tipo de pérdida auditiva, el 3 % (1/36) presentaba hipoacusia mixta, el 5 % (2/36), hipoacusia conductiva y, el 91 % (33/36), hipoacusia neurosensorial. En relación con la adquisición del lenguaje, el 3 % (1/36) presentaba hipoacusia perilingual, el 17 % (6/36), prelingual y, el 61 % (22/36), poslingual; del 19 % restante no pudo obtenerse información. En lo relacionado con el grado de compromiso auditivo, hubo una mayor frecuencia de hipoacusia profunda en el 36 % (13/36) de los casos, seguida por hipoacusia moderada en el 17 % (6/36) (cuadro 2). En la figura 1 se presenta la edad de inicio de la hipoacusia en los 36 individuos con esta condición.

**Cuadro 2:** Distribución del grado de compromiso auditivo (N=36) Tipo de hipoacusia según grado de compromiso

Tipo de hipoacusia según grado de compromiso	
Grado de compromiso	n
	
Leve	2
Leve-moderada	1
Moderada	6
Moderada-grave	1
Moderada en oído izquierdo y grave en oído derecho	2
Normal en oído derecho y moderada en oído izquierdo	1
Normal en oído izquierdo y profunda en oído derecho	1
Grave	2
Grave-profunda	1
Profunda	13
Sin datos	6

### Compromiso visual

**Figura 2:**
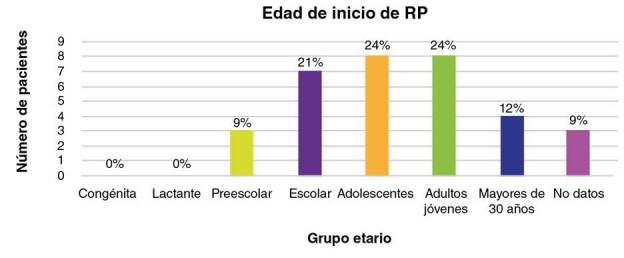
Distribución de la edad de inicio de la retinitis pigmentaria

**Figura 3:**
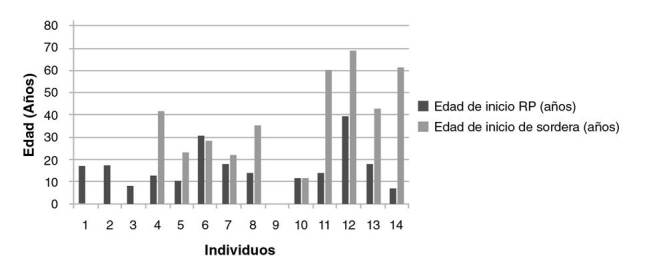
Edad de inicio de la retinitis pigmentaria y de la pérdida auditiva en casos de asociación de estas dos condiciones

El 76,4 % (42/55) de las personas analizadas presentaba algún compromiso ocular, y de estos, el 79 % (33/42) tenía retinitis pigmentaria, el 12 % (5/42), algún grado de disminución de la agudeza visual y, el 9 % (4/42), miopía. En la figura 2 se muestra la edad de inicio de la enfermedad de los 33 individuos con retinitis pigmentaria. Al comparar la edad de inicio de esta y de la sordera en los 14 individuos con la condición asociada a sordera, se observó que esta se había iniciado primero que la hipoacusia en todos los casos (figura 3).

Nota: Con respecto a los individuos 1 a 3, no se contó con los datos sobre la edad de inicio de la hipoacusia. El individuo 9 no informó la edad de inicio de los síntomas. RP: retinitis pigmentaria

En el cuadro 3 se presenta la caracterización de las 14 familias incluidas en el estudio con los síntomas identificados en cada caso. Se hallaron, además, dos individuos de sexo masculino de una misma familia con retinitis pigmentaria asociada a temblor distal y, en otra familia, un individuo con cofosis bilateral acompañada de microtia de grado I y un fenotipo llamativo con hipoplasia del tercio medio facial, paladar ojival, escoliosis y asteatosis, que no corresponden a un síndrome específico.

**Cuadro 3:** Caracterización fenotípica de las 14 familias incluidas en el estudio

Familias	Manifestaciones clínicas						Total afectados
	Hipoacusia	Retinitis pigmentaria	Retinitis pigmentaria más hipoacusia	Otro síntoma ocular	Otro síntoma ocular más hipoacusia	RP+ Temblor distal		
1	1	1	2	1		2	7
2	2	4			1		7
3	1		1				2
4	2		1		1		4
5		1	1	1			3
6	4		6	2			12
7	1	1		2			4
8		2	2				4
9	2		1				3
10	2	1					3
11	1	2					3
12			1				1
13			1				1
14			1				1

RP: retinitis pigmentaria

## Discusión

Uno de los objetivos del estudio era determinar una posible región geográfica donde estuviera concentrada la mayor cantidad de familias afectadas y, aunque la mayoría provenía del altiplano cundiboyacense (departamentos de Cundinamarca y Boyacá), también se encontraron familias de poblaciones lejanas. La concentración en esta región cercana a la capital se explicaría por la facilidad de acceso a los servicios de salud de alta complejidad, lo que produjo un sesgo en la recolección de datos.

Una de las principales causas de retinitis pigmentaria asociada a sordera es el síndrome de Usher, el cual se clasifica en tres tipos clínicos que varían según la edad de presentación de la condición, la gravedad de la hipoacusia, la progresión de las manifestaciones clínicas, y la asociación con otros signos y síntomas como el vértigo ^1^. Sin embargo, no toda hipoacusia con retinitis pigmentaria es un síndrome de Usher. Se han reportado casos de familias con diagnóstico clínico de síndrome de Usher atípico, que presentan variantes moleculares en genes relacionados con otro síndrome, y también, familias en las que puede sospecharse que tales manifestaciones clínicas se presentan de forma concomitante, pero independiente ^19^^,^^20^.

En las 14 familias analizadas en este estudio, había 23 mujeres y 32 hombres afectados por retinitis pigmentaria o hipoacusia con una relación hombre-mujer de 1,4:1. La edad de presentación fue muy variable; en el caso de la hipoacusia, los individuos refirieron diferentes edades de inicio (figura 1). De los 36 individuos con hipoacusia, tres (8 %) presentaban hipoacusia congénita, en tres (8 %), la pérdida auditiva se inició en la lactancia, en uno (3 %), en edad preescolar, en tres (8 %), en edad escolar, en cinco (14 %), cuando eran adultos jóvenes y, en otros cinco (14 %), después de los 30 años. En 16 (44 %) de los casos no fue posible obtener esta información.

La edad de presentación de la hipoacusia es un aspecto clave para hacer el diagnóstico de síndrome de Usher ^21^, pues se trata de una enfermedad congénita ^22^^,^^23^ bilateral, symmetric retinal degeneration that begins with night blindness and constricted visual fields (tunnel vision. En nuestro caso, la mayoría de los individuos de la población seleccionada no cumplía con este criterio diagnóstico, por lo cual no era posible diagnosticarles síndrome de Usher. Sin embargo, puede existir la posibilidad de que los pacientes con hipoacusia congénita leve se diagnosticaran tardíamente, durante el primer año de vida o después de evidenciarse el trastorno del habla ^24^. Por otra parte, en el síndrome de Usher de tipo III, se ha descrito la aparición de hipoacusia en la segunda y cuarta décadas de la vida ^25^^,^^26^, lo cual puede dificultar el diagnóstico temprano.

En cuanto a la progresión de la hipoacusia, que es un criterio diagnóstico del síndrome de Usher de tipo III ^27^, se logró valorar periódicamente con audiometría y logoaudiometría a 16 individuos y se encontró que 12 de ellos (75 %) cursaban con hipoacusia progresiva, en cuatro de los casos iniciada en la primera década de vida, en seis en la segunda y en dos en la tercera. Existe la posibilidad de que la hipoacusia se iniciara a edades más tempranas, pero dada la dificultad de los pacientes para detectar las pérdidas auditivas, estas se habrían reportado a edades más avanzadas.

De los 36 individuos con hipoacusia, el 78 % presentaba compromiso bilateral y, de estos, el 89 % fue diagnosticado con hipoacusia neurosensorial, el 4 % con la forma mixta y el 7 % con hipoacusia conductiva, que no puede descartarse como secundaria a múltiples episodios infecciosos de otitis media aguda en la infancia, no reportados por el afectado. En estos casos, debe tenerse especial cuidado al hacer el diagnóstico ya que, en un paciente con retinitis pigmentaria asociada con hipoacusia, esta asociación puede confundirse con el síndrome de Usher. Ante esta dificultad diagnóstica, es necesario hacer una anamnesis minuciosa y elaborar un árbol genealógico que revele los familiares afectados y sus edades, así como la edad de presentación y la consanguinidad.

En cuanto a la relación de la hipoacusia con la adquisición del lenguaje en los 36 individuos, se observó que la hipoacusia fue perilingual en el 3 % ^1^, prelingual en el 17 % ^6^, y poslingual en el 61 % ^22^; el promedio de edad de presentación fue de 21 años, por lo cual muchos de los individuos conservaban el lenguaje expresivo ^28^.

Tanto el síndrome de Usher de tipo I como el de tipo II, se caracterizan por hipoacusia prelingual con la subsecuente alteración del habla ^29^. Por otro lado, en el síndrome de Usher de tipo III, se ha descrito hipoacusia poslingual de leve a grave. Ahora bien, del total ^22^ de individuos con hipoacusia neurosensorial bilateral poslingual, el 72,7 % ^16^ presentaba hipoacusia moderada a grave. Además, la progresión también parece ser variable, pues se reporta en algunos pacientes y en otros no.

En general, en el tipo III todo parece ser variable, incluso la aparición de la retinitis pigmentaria puede darse en la primera o segunda década de la vida y, también, varía la presencia de alteración vestibular. Todo ello dificulta el diagnóstico basado en criterios clínicos. En este grupo, se seleccionaron aquellos con diagnóstico de retinitis pigmentaria y, en 13 (81 %) de ellos, fue necesaria la prueba molecular para confirmar la sospecha de síndrome de Usher de tipo III.

Al analizar todos los afectados por hipoacusia sola o asociada con otra manifestación clínica, el 76 % de los Individuos con hipoacusia y el 93 % de aquellos con retinitis pigmentaria y sordera, tenían la forma poslingual con inicio en la edad adulta, lo cual tampoco corresponde a un diagnóstico de síndrome de Usher típico.

Un grupo de pacientes que presentaba miopía como único síntoma ocular de inicio en la tercera década de la vida, también tenía hipoacusia poslingual. En estos se descartó el síndrome de sordera asociado con miopía, ya que en esta condición la miopía suele ser mayor de 6 dioptrías y se presenta en la infancia ^30^^,^^31^, lo cual no fue el caso en los individuos de este estudio.

En el caso de los 33 individuos con retinitis pigmentaria, se reportaron diferentes edades de inicio, que variaron desde la edad preescolar hasta la adultez ^32^. No se encontraron casos de la forma congénita ni de inicio en la lactancia, como se observa en la figura 2, pues 8 (24 %) individuos presentaron la retinitis pigmentaria siendo adultos jóvenes y 8 (24 %) en la adolescencia, 7 (21 %) de ellos en edad escolar, 4 en la adultez (12 %) y 3 en preescolar (9 %). En los estudios consultados, la edad típica de inicio del síndrome de Usher de tipos I y II se registró en las primeras dos décadas de la vida ^33^. En un estudio de tres familias de la población de Newfoundland, hubo individuos que presentaron la retinitis pigmentaria después de la tercera década ^34^. Aunque es posible que fuera realmente tardía, también cabe considerar que se hubiera iniciado inicio en edades más tempranas, pero no se hubiera detectado por su progresión lenta, hasta llegar al punto en que los síntomas se hicieron evidentes. Para determinar esto con mayor certeza, se requeriría la valoración periódica desde el nacimiento mediante herramientas oftalmológicas que permitan un monitoreo más sensible de la enfermedad para, así, detectar cambios retinianos leves que puedan manejarse desde temprano, con el fin de ralentizar su progresión ^35^.

En el marco de proyectos previos de investigación de nuestro grupo y de otros, se ha observado que, en muchos individuos con retinitis pigmentaria, es frecuente encontrar el antecedente de miopía, la cual sí suele evidenciarse en edades tempranas. Ello no significa que toda retinitis pigmentaria se acompañe de miopía, ni que toda miopía derive en ella ^36^. El 46 % de los individuos analizados en el presente estudio coincidió en afirmar que presentaba una disminución de la agudeza visual diagnosticada tempranamente como miopía. Con el fin de establecer una posible asociación entre estos dos diagnósticos, se han realizado estudios de correlación entre el genotipo y el fenotipo en pacientes con miopía y retinitis pigmentaria, y se han encontrado mutaciones en los genes *RP1, RPGR* y *RP2*, los cuales se relacionan con retinitis pigmentaria no sindrómica y miopía ^37^.

En cuanto a los individuos del presente estudio, es necesario hacer análisis moleculares para identificar posibles variantes asociadas con el fenotipo. Se ha planteado la teoría de que los cambios iniciales leves en el epitelio pigmentario de la retina, que se presentan en la distrofia de conos y bastones, pueden alterar la captación de señales luminosas y, por lo tanto, confundirse con un defecto de la refracción, en este caso, la miopía ^38^^,^^39^. También, se ha expuesto la hipótesis de que el depósito de pigmento conlleva degeneración de los fotorreceptores en modelos de ratón y se ha planteado que el compromiso de los fotorreceptores puede alterar el metabolismo de la dopamina asociado con la propensión a la miopía ^40^.

Sin embargo, en nuestra experiencia y en el seguimiento de esos casos, lo que se ha observado es que el paciente presenta inicialmente los signos clásicos de una miopía acusada, como la nictalopía, y varios años después comienzan a evidenciarse las espículas de hueso y el fenotipo correspondientes a la retinitis pigmentaria ^41^. Esta puede ser muy difícil de sospechar en sus estadios muy iniciales porque las manifestaciones clínicas pueden superponerse y porque, en algunos casos, el sitio inicial de compromiso de la retina puede ser la periferia ^37^^,^^42^^,^^43^.

Por otro lado, llama la atención que el 39 % ^13^ de los 33 individuos con retinitis pigmentaria refirió como primera manifestación ocular la nictalopía: seis en la primera década de vida, cinco en la segunda, uno en la tercera y uno en la sexta. Aunque esta podría deberse a la variabilidad de expresión de la retinitis pigmentaria ^44^, no se descarta que en quienes refieren nictalopía después de la segunda década de vida, ello se deba a que no pudieron identificar el síntoma de forma temprana; es usual que otras personas sean quienes observen en los sujetos afectados cambios en su desplazamiento en la penumbra, o caídas frecuentes en los niños. En estos casos, la demora en el diagnóstico se debe a que los pacientes consultan tardíamente, cuando ya hay otros síntomas.

En la población analizada, se encontró que 14 (25,5 %) individuos presentaban retinitis pigmentaria asociada a hipoacusia. Según lo recopilado en el registro médico y en el momento de ingreso al estudio, todos los sujetos con estas dos condiciones presentaron primero la retinitis, entre la segunda y la tercera décadas de la vida, y después el compromiso auditivo, lo que contradice lo descrito en la historia natural del síndrome de Usher ^45^ en el cual la hipoacusia es congénita y, por lo tanto, se sospecha y diagnostica más temprano, ya sea por tamizaje neonatal o por el seguimiento del desarrollo del lenguaje en los primeros años de vida.

En ninguna de las familias se pudo determinar un patrón claro de herencia autosómica recesiva, a pesar de que se reportó consanguinidad en el 29 % (16 individuos) en seis de las familias. El 71 % restante no presentaba consanguinidad y no pertenecía a comunidades endogámicas. Además, no se logró determinar una región geográfica común en los ancestros de las 14 familias. Considerando lo expuesto y dado que la herencia autosómica recesiva es el patrón de herencia característico del síndrome de Usher, a pesar de que los individuos analizados en este estudio tenían retinitis pigmentaria asociada a hipoacusia neurosensorial bilateral, no presentaban claramente un síndrome de Usher típico.

La variabilidad de expresión y la heterogeneidad genética dificultan el diagnóstico clínico de los individuos afectados. En la literatura se presentan casos en los cuales, aunque están presentes los criterios que definen un posible diagnóstico clínico de síndrome de Usher, el estudio molecular identifica variantes patogénicas asociadas con otros síndromes ^26^. También, se reportan individuos que no cumplen con los todos los criterios clínicos para un diagnóstico de síndrome de Usher y que son clasificados como “Usher atípicos” porque son portadores de variantes patogénicas en genes asociados con esta condición ^46^.

Khateb, *et al*. ^16^, hicieron la secuenciación exómica y genómica de cinco individuos de tres familias de judíos yemeníes que presentaban degeneración retiniana y pérdida auditiva neurosensorial. Describieron dos hermanos con compromiso de retina después de los 50 años, atrofia en anillo del nervio óptico, acumulación de pigmento en la retina y posterior hipoacusia neurosensorial progresiva. En la segunda familia descrita, los hijos eran cuatro, un hombre y tres mujeres, dos de ellas afectadas por retinitis pigmentaria e hipoacusia aparecida en diferentes edades, incluso, en una de ellas el compromiso visual apareció en la segunda década de la vida. En la tercera familia, encontraron a dos hermanas afectadas por hipoacusia neurosensorial progresiva, una de ellas desde la infancia, con aparición posterior de la retinitis pigmentaria.

Las familias descritas son similares a las de nuestro estudio en cuanto a las edades de presentación atípicas de cada una de las manifestaciones clínicas, la presentación inicial de la retinitis pigmentaria y la aparición de hipoacusia progresiva después de la sexta década de la vida, sin compromiso vestibular. En estos casos, existe una alta sospecha de Usher atípico; sin embargo, no se pueden descartar otras causas, como ha sucedido en algunos estudios en los que se hallaron variantes patogénicas asociadas con enfermedad lisosómica mediante secuenciación de próxima generación ^5^^,^^34^.

Katheb, *et al*. ^19^, reportaron una familia consanguínea iraní de origen judío, en la que siete sujetos estaban afectados por diversos grados de compromiso auditivo y visual debido a degeneración retiniana leve en unos, y grave y temprana en otros. A pesar de la consanguinidad informada y del estudio de segregación, a los autores no les fue posible determinar un patrón de herencia específico en función del árbol genealógico.

En los árboles genealógicos de las familias del presente estudio, fue llamativo encontrar generaciones con individuos afectados solo por retinitis pigmentaria o solo por sordera, algunos de ellos con un patrón de herencia autosómico dominante en las primeras tres generaciones, ya fuera de retinitis pigmentaria o de hipoacusia, en tanto que, en la generación del probando, se encontró a un sujeto con esta condición acompañada de hipoacusia neurosensorial, lo cual no permite definir la enfermedad como un síndrome de Usher típico. En estos casos, es esencial identificar el gen o los genes implicados para, así, aclarar el diagnóstico clínico diferencial.

En algunos estudios, se ha reportado un fenotipo de hipoacusia neurosensorial aislada en individuos con mutaciones en los genes asociados con el síndrome de Usher, como es el caso de *MYO7A* y *CHDH23*, en los que algunos tipos de mutaciones específicas en el mismo gen determinan un fenotipo diferente en los individuos afectados: las mutaciones con cambio de sentido o con cambio del marco de lectura causan sordera no sindrómica ^47^, en tanto que, aquellas que generan proteína truncada dan lugar a un fenotipo de síndrome de Usher ^48^^,^^49^. Esto puede estar presentándose en nuestra población de estudio, en la que diferentes tipos de mutaciones en un mismo gen probablemente han resultado en la afección de diversos dominios y en la generación de isoformas, lo cual se asocia con las diferentes presentaciones clínicas de la enfermedad en cada una de las familias.

Se han informado casos de distrofia de retina más retinitis pigmentaria pertenecientes a familias con más de un miembro afectado por hipoacusia neurosensorial, en quienes no se logra inicialmente identificar variantes de genes asociados con el síndrome de Usher, pero sí, al ampliar su estudio, de variantes de otros genes ^46^.

En el presente estudio se encontraron dos hombres de una misma familia que presentaban retinitis pigmentaria asociada con temblor distal. En el momento del estudio, ninguno tenía ataxia, ni antecedentes de hipotonía, ni otra alteración visual, como tritanopatía u oftalmoparesia, que permitiera hacer el diagnóstico de ataxia espinocerebelosa (*Spinocerebellar Ataxia*, SCA). En esta familia, será necesario caracterizar los síntomas neurológicos para hacer el diagnóstico preciso y descartar una SCA2 ^50^ o una SCA7 ^51^^,^^52^. En otra familia, se encontró un hombre con diagnóstico de cofosis bilateral, microtia de grado III y frente amplia, quien requiere seguimiento de genética clínica y un más completo estudio etiológico. Por último, no se encontraron individuos con alteración de la función vestibular en la población estudiada.

Se ha reportado que, a pesar de que muchos fenotipos concuerdan con los criterios diagnósticos de alguno de los tres tipos de síndrome de Usher, un número significativo de ellos no cumplen con todos los criterios. Por lo tanto, se ha recomendado establecer guías más precisas de nomenclatura de los individuos con estos genotipos y fenotipos atípicos ^53^.

En resumen, este estudio resalta la importancia de una caracterización fenotípica completa y detallada, no solamente del caso índice, sino también de los familiares. La evaluación completa del fenotipo retiniano ayuda a confirmar si se trata de una retinitis pigmentaria típica, que encaje en los criterios del síndrome de Usher. En tanto que la evaluación precisa y la correcta caracterización de la función vestibular y del fenotipo auditivo, así como la presencia de otras manifestaciones indicativas de una forma sindrómica similar al síndrome de Usher, son indispensables para apoyar el diagnóstico en cada caso. Por otra parte, la evaluación de los antecedentes familiares puede esclarecer mucho más el diagnóstico al evidenciar otro tipo de manifestaciones que pueden estar heredándose de forma independiente en las familias, lo que descartaría el diagnóstico de síndrome de Usher. Es fundamental el estudio familiar en los casos en los que la clínica no concuerda con las características del síndrome de Usher típico. En los casos de retinitis pigmentaria asociada a sordera, el diagnóstico clínico acertado permite orientarse hacia los análisis moleculares y, así, establecer un diagnóstico diferencial.

Se enfatiza la necesidad de elaborar guías de nomenclatura para los individuos con estos hallazgos atípicos, que permitan orientar a médicos e investigadores en el correcto manejo de estos casos.

Como limitaciones de este estudio, deben mencionarse el sesgo de memoria, frecuente en la variable de edad de inicio de los diferentes síntomas; y además, en la recolección de datos, principalmente aquellos sobre la posible afección en algunos individuos de la familia, así como en la recolección completa de la historia natural de las enfermedades de los pacientes.

## References

[1] Malm E, Ponjavic V, Möller C, Kimberling WJ, Andréasson S (2011). Phenotypes in defined genotypes including siblings with Usher syndrome. Ophthalmic Genet.

[2] Kaplan HJ, Wang W, Dean DC (2017). Restoration of cone photoreceptor function in retinitis pigmentosa. Transí Vis Sci Technol.

[3] Pakarinen L, Tuppurainen K, Laippala P, Mántyjárvi M, Puhakka H (1997). The ophthalmological course of Usher syndrome type III. Ophthalmic Lit.

[4] Rabin J, Houser B, Talbert C, Patel R (2017). Measurement of dark adaptometry during ISCEV standard flash electroretinography. Doc Ophthalmol.

[5] Puffenberger EG, Jinks RN, Sougnez C, Cibulskis K, Willert RA, Achilly NP (2012). Genetic mapping and exome sequencing identify variants associated with five novel diseases. PLoS ONE.

[6] Parmeggiani F, Sorrentino F, Ponzin D, Barbara V, Ferrari S, Di Lorio E (2011). Retinitis pigmentosa: Genes and disease mechanisms. Curr Genomics.

[7] Berson EL, Rosner B, Simonoff E (1980). Risk factors for genetic typing and detection in retinitis pigmentosa. Am J Ophthalmol.

[8] Cummings C, Fredrickson J, Harker L (2005). Otolaryngology: Head and neck surgery.

[9] Boughman JA, Vernon M, Shaver KA (1983). Usher syndrome: Definition and estimate of prevalence from two high-risk populations. J Chronic Dis.

[10] Cortés RA, Cenjor C, Ayuso C (2004). Síndrome de Usher: aspectos clínicos, diagnósticos y terapéuticos.

[11] Tamayo ML, Bernal JE, Tamayo GE, Frías JL, Alvira G, Vergara O (2008). Usher syndrome: Results of a screening program in Colombia. Clin Genet.

[12] Leal GL, Moyano NG, Tamayo ML (2010). Definición de subtipos del síndrome de Usher en población colombiana. Medicina (Mex).

[13] Tamayo ML, Bernal JE, Tamayo GE, Frías JL (1992). Study of the etiology of deafness in an institutionalized population in Colombia. Am J Med Genet.

[14] Khan KN, El-Asrag ME, Ku CA, Holder GE, McKibbin M, Arno G (2017). Specific alleles of CLN7 / MFSD8, a protein that localizes to photoreceptor synaptic terminals, cause a spectrum of nonsyndromic retinal dystrophy. Investig Opthalmology Vis Sci.

[15] Neuhaus C EisenbergerT, Decker C Nagl S, Blank C Pfister M (2017). Next-generation sequencing reveals the mutational landscape of clinically diagnosed Usher syndrome: Copy number variations, phenocopies, a predominant target for translational read-through, and PEX26 mutated in Heimler syndrome. Mol Genet Genomic Med.

[16] Khateb S, Kowalewski B, Bedoni N, Damme M, Pollack N, Saada A (2018). A homozygous founder missense variant in arylsulfatase G abolishes its enzymatic activity causing atypical Usher syndrome in humans. Genet Med.

[17] Trouillet A, Dubus E, Dégardin J, Estivalet A, Ivkovic I, Godefroy D (2018). Cone degeneration is triggered by the absence of USH1 proteins but prevented by antioxidant treatments. Sci Rep.

[18] Orejas J, Rico J (2013). Hipoacusia: identificación e intervención precoces. Pediatría Integral.

[19] Khateb S, Zelinger L, Mizrahi-Meissonnier L, Ayuso C, Koenekoop RK, Laxer U (2014). A homozygous nonsense CEP250 mutation combined with a heterozygous nonsense C2orf71 mutation is associated with atypical Usher syndrome. J Med Genet.

[20] Namburi P, Ratnapriya R, Khateb S, Lazar CH, Kinarty Y, Obolensky A (2016). Bi-allelic truncating mutations in CEP78, encoding centrosomal protein 78, cause cone-rod degeneration with sensorineural hearing loss. Am J Hum Genet.

[21] Ren SM, Wu QH, Chen YB, Jiao ZH, Kong XD (2021). Variation analysis of genes associated with Usher syndrome type 1 in 136 Chinese deafness families. Zhonghua Er Bi Yan Hou Tou Jing Wai Ke Za Zhi.

[22] Lentz J, Keats B, Adam MR Ardinger HH, Pagon RA (2016). GeneReviews®.

[23] Lentz J, Keats BJ, Adam MR Ardinger HH, Pagon RA (2016). GeneReviews®.

[24] Delgado JJ, Grupo Previnfad/PAPPS infancia y adolescencia (2011). Detección precoz de la hipoacusia infantil. Rev Pediatr Aten Primaria.

[25] Mathur R Yang J (2015). Usher syndrome: Hearing loss, retinal degeneration and associated abnormalities. Biochim Biophys Acta.

[26] EisenbergerT Slim R, MansourA Nauck M, Nürnberg G Nürnberg R (2012). Targeted next- generation sequencing identifies a homozygous nonsense mutation in ABHD12, the gene underlying PHARC, in a family clinically diagnosed with Usher syndrome type 3. Orphanet J Rare Dis.

[27] Bauer C, Jenkins H, Flint PW, Haughey BH, Lund VJ, Niparko JK, Richardson MA, Robbins KT (2015). Cummings Otolaryngology Head & Neck Surgery.

[28] Hildebrand MS, Husein M, Smith RJ, Flint PW, Haughey BH, Lund VJ, Niparko JK, Richardson MA, Robbins KT (2015). Cummings Otolaryngology Head & Neck Surgery.

[29] López G, Gélvez NY, Tamayo M (2011). Frecuencia de mutaciones en el gen de la usherina (USH2A) en 26 individuos colombianos con síndrome de Usher, tipo II. Biomédica.

[30] Ordóñez J, Tekin M, Adam MR Ardinger HH, Pagon RA (2017). Deafness and myopia syndrome.

[31] Shearer AE, Hildebrand MS, Smith RJ, Adam MR Ardinger HH, Pagon RA (2017). GeneReviews®.

[32] Nagase Y, Kurata K, Hosono K, Suto K, Hikoya A, Nakanishi H (2018). Visual outcomes in Japanese patients with retinitis pigmentosa and Usher syndrome caused by USH2A mutations. Semin Ophthalmol.

[33] Testa F, Melillo P, Bonnet C, Marcelli V, De Benedictis A, Colucci R (2017). Clinical presentation and disease course of Usher syndrome because of mutations in MY07A or USH2A. Retina.

[34] Pater JA, Green J, O’Rielly DD, Griffin A, Squires J, Burt T (2019). Novel Usher syndrome pathogenic variants identified in cases with hearing and vision loss. BMC Med Genet.

[35] Sun LW, Johnson RD, Langlo CS, Cooper RF, Razeen MM, Russillo MC (2016). Assessing photoreceptor structure in retinitis pigmentosa and Usher syndrome. Investig Opthalmology Vis Sci.

[36] Ayuso C, Millan JM (2010). Retinitis pigmentosa and allied conditions today: A paradigm of translational research. Genome Med.

[37] Chassine T, Bocquet B, Daien V, Ávila-Fernández A, Ayuso C, Collin RW (2015). Autosomal recessive retinitis pigmentosa with RP1 mutations is associated with myopia. Br J Ophthalmol.

[38] Zhang Y, Wildsoet CF, Klettner AK, Dithmar S (2020). Retinal pigment epithelium in health and disease.

[39] Klettner AK, Dithmar S (2020). Retinal pigment epithelium in health and disease.

[40] Park H, Tan CC, Faulkner A, Jabbar SB, Schmid G, Abey J (2013). Retinal degeneration increases susceptibility to myopia in mice. Mol Vis.

[41] Gregory-Evans K, Pennesi ME, Weleber RG, Ryan SJ, Sadda SR, Schachat AP (2013). Retina.

[42] Sánchez-Tocino H, Diez-Montero C, Villanueva-Gómez A, Lobo-Valentín R, Montero-Moreno JA (2019). Phenotypic high myopia in X-linked retinitis pigmentosa secondary to a novel mutation in the RPGR gene. Ophthalmic Genet.

[43] Lu Y, Sun X (2021). Retinitis pigmentosa sine pigmento masqueraded as myopia: A case report (CARE). Medicine (Baltimore).

[44] Dad S, Rendtorff ND, Tranebjaerg L, Gronskov K, Karstensen HG, Brox V (2016). Usher syndrome in Denmark: Mutation spectrum and some clinical observations. Mol Genet Genomic Med.

[45] García-García G, Aparisi MJ, Jaijo T, Rodrigo R, León AM, Ávila-Fernández A (2011). Mutational screening of the USH2A gene in Spanish USH patients reveals 23 novel pathogenic mutations. Orphanet J Rare Dis.

[46] Bashir R, Fatima A, Naz S (2010). A frameshift mutation in SANS results in atypical Usher syndrome. Clin Genet.

[47] Adam M, Ardinger H, Pagon R, Wallace S, Bean L, Stephens K (1998). GeneReviews®.

[48] Hildebrand M, Thorne N, Bromhead C, Kahrizi K, Webster J, Fattahi Z (2010). Variable hearing impairment in a DFNB2 family with a novel MY07A missense mutation. Clin Genet.

[49] Faundes V, Pardo RA, Castillo-Taucher S (2012). Genética de la sordera congénita. Med Clínica.

[50] Antenora A, Rinaldi C, Roca A, Pane C, Lieto M, Saccà F (2017). The multiple faces of spinocerebellar ataxia type 2. Ann Clin Transí Neurol.

[51] Rossi M, Pérez-Lloret S, Doldan L, Cerquetti D, Balej J, Millar-Vernetti P (2014). Autosomal dominant cerebellar ataxias: A systematic review of clinical features. Eur J Neurol.

[52] Giocondo F, Curdo G (2018). Spinocerebellar ataxia: A critical review of cognitive and socio- cognitive deficits. Int J Neurosci.

[53] Nolen R, Hufnagel R, Friedman T, Turriff A, Brewer C, Zalewski C (2020). Atypical and ultra- rare Usher syndrome: A review. Ophthalmic Genet.

